# Renal transplant patient acceptance of a self-management support system

**DOI:** 10.1186/s12911-017-0456-y

**Published:** 2017-05-08

**Authors:** Wenxin Wang, Céline L. van Lint, Willem-Paul Brinkman, Ton J. M. Rövekamp, Sandra van Dijk, Paul J. M. van der Boog, Mark A. Neerincx

**Affiliations:** 1 Interactive Intelligence Group, Delft University of Technology, Mekelweg 4, 2628 CD Delft, The Netherlands; 2 TNO, the Hague, The Netherlands; 3 Department of Nephrology, Leiden University Medical Center, Leiden, The Netherlands; 4 Faculty of Social and Behavioral Sciences, Health, Medical and Neuropsychology Unit, Leiden University, Leiden, The Netherlands

**Keywords:** Technology acceptance, e-health, Renal transplant patient, Self-management, Survey

## Abstract

**Background:**

Self-management support systems (SMSS) have been proposed for renal transplant patients to increase their autonomy and reduce the number of hospital visits. For the design and implementation of such systems, it is important to understand factors influencing patients’ acceptance of a SMSS. This paper aims to identify these key factors.

**Methods:**

From literature, possible factors and related questionnaire items were identified. Afterwards, focus groups with experts and patients were conducted to adapt the items to the application domain. To investigate acceptance of a SMSS and the influencing factors, fifty renal transplant patients answered the questionnaire before and after using the SMSS for 4 months.

**Results:**

All the questionnaire constructs had a satisfactory or higher level of reliability. After using the SMSS for 4 months, trust and performance expectancy could explain part of the variation in behavioural intention of using the SMSS, but not beyond the explanation given by patients’ affect towards the system, which accounted for 26% of the variance.

**Conclusions:**

We anticipate that in future caregivers implementing a SMSS will benefit from taking steps to improve patients’ affect as this was found to correlate with patients use intention.

**Trial registration:**

The study was registered in ToetsingOnline, a registry held by the Dutch Central Committee on Research Involving Human Subjects. The registration number is NL33387.058.11, and the date of registration is 31st July 2012.

**Electronic supplementary material:**

The online version of this article (doi:10.1186/s12911-017-0456-y) contains supplementary material, which is available to authorized users.

## Background

Chronic kidney disease (CKD) is regarded as a major public health problem [1]. In the last stage of this disease, referred to as end-stage renal disease (ESRD), the preferred treatment is renal transplantation. Mortality rates for these patients are less than half compared to patients receiving dialysis treatment [2]. In addition, patients gain more freedom and energy from a successful kidney transplantation than from dialysis [3]. After kidney transplantation, however, patients need to adhere to a strict medication regimen and are followed-up frequently to monitor for signs of graft dysfunction or comorbidities. Kidney transplant patients are therefore still considered to have a chronic disease.

Self-management, the process of managing symptoms, treatment, physical and psychosocial consequences by patients themselves in daily life, has been proposed to be useful when dealing with chronic illness [4]. Self-management support systems (SMSSs) can help to increase the level of self-management [5]. These systems aim at empowering patients by giving them more control of their care process and daily activities and thereby increasing their autonomy [5].

SMSSs have already been successfully used in the health domain to support healthy behaviours, and reports indicate that people are capable of using them. Examples include an internet-based diabetes self-management and support system [6], and systems to manage physical activities [7–9], fruit and vegetables consumption [8], and medication intake [9].

### Need for a specific model

Besides users’ capability, their willingness, i.e. acceptance of using a SMSS, is also important. Several theories and models have been proposed to explain users’ acceptance of information technology (IT) or information systems. These theories explore the underlying factors of users’ acceptance, so that designers and organisations can anticipate on them to improve system acceptance. Both generic and specific models have been developed. The theory of reasoned action (TRA) [10], the theory of planned behaviour (TPB) [11], and the technology acceptance model (TAM) [12] are generic models formulated to apply across domains. Specific models, which are often derived from generic models, have been formulated for specific domains, such as models for Internet commerce [13, 14], online gaming [15], and mobile commerce [16].

In the area of health informatics and chronic diseases, understanding the acceptance of a SMSS could benefit from a specific model with its own unique set of factors and values, as the use of the technology may influence patients’ health and lives: people may be more concerned and reserved to use an SMSS. For example, interviews with diabetic patients about a SMSS for their insulin therapy showed that emotional aspects were important, such as being embarrassed to inject insulin in public or fear of hypoglycaemia when increasing insulin dose [17]. For patients with depression or with an increased risk of cardiovascular problems, the level of interest in using a telehealth application was found to be related to confidence and perceived advantages and disadvantages of the application [18]. Furthermore, studies of internet-based testing for sexually transmitted diseases [19] and the use of personal electronic health records and secure messaging [20] put forward internet and technology usage, health care access, provider satisfaction, interactions between environmental factors, and interactions between patient activation and tool empowerment potential as key factors determining people’s use of SMSSs. Arning and Karsh have also noticed that the current IT acceptance models were insufficient to understand patients [21, 22], and various researchers have worked on determining relevant factors that explain patients’ behavioural intention to use eHealth technology [22–25].

Renal transplant patients, however, might be at more risk than the previous examples of chronic patients, as rejection can occur acutely with the risk of losing the transplanted kidney. Although other domains such as office applications or e-commerce, even the eHealth domain in general, have received substantial research attention, less is known about patient acceptance of a SMSS in general and more specifically, the acceptance of a SMSS by renal transplant patients.

### Objective

To better understand the renal transplant patients and their acceptance of using a SMSS, this paper studies their intention of using a SMSS and the underlying factors that explain this use intention. This understanding would allow system designers and health program managers to direct their attention and effort effectively and efficiently.

## Literature review

**Fig. 1:**
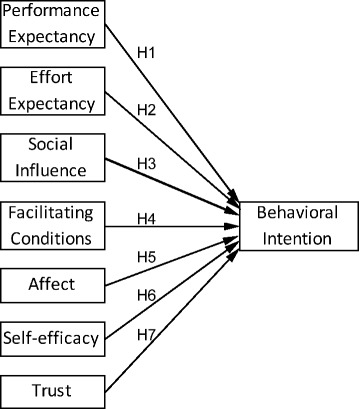
Renal transplant patient technology acceptance model

The most well-known models or theories that have been used to explain peoples’ acceptance of technology are the theory of reasoned action (TRA) [10], the theory of planned behaviour (TPB) [11], the technology acceptance model (TAM) [12], and their extensions, such as TAM2 [26], the unified theory of acceptance and use of technology (UTAUT) [27], and TAM3 [28]. These models are used widely, and their coefficient of determination (*R* ^2^) ranged from 17 to 70%. In other words, the factors in these models can explain this amount of variation between people’s intentions to use information technology [27]. *R* ^2^ is calculated by the squaring the correlation between the predicted behavioural intention by the model and the actual behavioural intention reported by the individuals. Further meta-analysis and review showed that TAM and its extensions are valid and robust, but more variables should be integrated to enhance the explained variance regarding the acceptance and use of technology [29, 30]. These models are generic as they were aimed to apply across domains, and did not consider the different context of specific domains, such as eHealth or eCommerce. These generic theories and models have been used to formulate a renal transplant patient technology acceptance (RTPTA) model for a SMSS (Fig. 1). In the remainder of this section, each determinant in the model is defined and provided with the theoretical justification.

### Performance expectancy

Performance expectancy (PE) is adapted from UTAUT [27] and is defined here as the degree to which renal patients believe that using the system will help them attain gains or make losses with the performance of their health management. It investigates if participants expect that the system can help them with monitoring their health. PE is strongly related to the perceived usefulness construct in TAM [31]. In many studies, PE has been shown to be one of the strongest predictors of behavioural intention [23, 24, 27] and it has been used in the health informatics domain before, for example by Ahadzadeh [23] and Beenkens [24]. This leads to the first hypothesis:

*H1: Performance expectancy positively correlates with patients’ intention to use the SMSS.*

### Effort expectancy

Effort expectancy (EE) is defined as the degree of ease associated with the use of the system [27], e.g., whether patients experience any difficulties using the system. Perceived ease of use (PEOU) in TAM is a theoretically similar construct and is mainly found an effective predictor for peoples’ use intention when they are new to a technology [27]. EE has been shown to have a significant effect on patients’ intention of using an e-health service [24]. This leads to the second hypothesis:

*H2: Effort expectancy positively correlates with patients’ intention to use the SMSS.*

### Social influence

Social influence (SI) is also adapted from UTAUT [27] and is defined here as the degree to which renal patients perceive that important others believe they should use the system. It refers to what people in the patients’ environment think of using the system. TRA, TPB, TAM2, and TAM3 refer to this construct as subjective norm [11, 26, 28, 32]. Venkatesh et al. were unable to find SI as an effective predictor for voluntary technology use [27]. However, they did find it to be an effective predictor in a compulsory use context, for example when the working environment requires using that specific software application; but only at a stage where people had limited use experience. In the context of health-management, patients’ usage of a technology is often voluntary, the decision on whether or not using a system might be influenced by health-providers, family members, or fellow patients. Kim and Park have reported subjective norm to have a strong indirect association with patients’ behavioural intention of using health information technology via perceived usefulness [25]. This leads to the third hypothesis:

*H3: Social influence positively correlates with patients’ intention to use the SMSS.*

### Facilitating conditions

The factor referred to as facilitating conditions (FC) is often put forward as an effective predictor [27, 33]. In the current model, FC is defined as the degree to which renal patients believe that there are objective factors available in their environment to support their use of the system [27]. Examples of these objective factors include a computer that is appropriate for use of the system, and the availability of supporting others who can help to use the system if needed. Studies have reported mixed outcomes concerning the relevance of facilitating conditions for behavioural intention [27, 34, 35]. In the eHealth domain, however, facilitating conditions are considered an important predictor of patients’ acceptance [22]. This leads to the fourth hypothesis:

*H4: Facilitating conditions positively correlate with patients’ intention to use the SMSS.*

### Affect

Affect (AF) is defined as the renal patients’ overall affective reaction towards using the system. It addresses whether individuals find it pleasant to use the system. TRA, TBP, TAM nor UTAUT include the emotional reaction in performing the intended behaviour directly in their model. Instead, emotional outcomes are only indirectly included in the models as attitude towards the intended behaviour [12, 32, 36, 37]. Others have argued for the inclusion of affect as a separate construct because one’s liking of a technology could influence his or her actual usage of this technology [38]. For example, computer games are used in healthcare domain because they have the advantage of entertaining people in otherwise painful or boring health promoting processes [39]. Anxiety, as the opposite of liking, is expected to negatively influence system use [38]. In fact, affect has been found to be a predicting factor for general IT usage [38]. This leads to the fifth hypothesis:

*H5: Affect positively correlates with patients’ intention to use the SMSS.*

### Self-efficacy

Self-efficacy (SE) is a key factor in predicting people’s behaviour as it determines if they will initiate certain behaviour, how much effort they will spend on it, and how they will cope with potential obstacles [40]. In the current model, SE is defined as the degree to which renal patients judge themselves capable of using the system to manage their health, which is in line with Compeau and Higgins [38]. The concerning items address if patients think they can handle the system. So far, results concerning the role of self-efficacy in technology acceptance have been mixed. Venkatesh et al., for example, left out self-efficacy in the UTAUT model because they failed to find a stable association over time between self-efficacy and behavioural intention [27]. Others, however, do report self-efficacy beliefs as a significant precursor to information technology use [41, 42]. In the health informatics domain, however, self-efficacy was found to be indirectly linked with behavioural intention by influencing perceived usefulness and perceived ease of use [25]. This leads to the sixth hypothesis:

*H6: Self-efficacy positively correlates with patients’ intention to use the SMSS.*

### Trust

Trust (TR) is defined as the degree to which patients believe that using the system will occur in a safe and reliable manner, consistent with their expectations of the health management task [13]. The latter is important because using any system does not mean that the patients themselves will always be safe, but that the system will run in a safe and reliable way. Participants are therefore asked how trustworthy they find the system. Although trust is not included in the generic models, it has been included in extensions of these models, for example as an extension of TAM regarding Internet shopping [43, 44]. In this case, people are concerned about losing their money, which might stop them from making online purchases. Similarly in the health informatics domain, various trust aspects have been identified, including personal technical insecurity, perceived threat, and perceived health risk [23–25]. Renal patients’ trust in a SMSS is therefore suggested to influence their willingness to use such a system. This leads to the seventh hypothesis:

*H7: Trust positively correlates with patients’ intention to use the SMSS.*

### Behavioural intention

Behavioural intention (BI) is defined as the degree to which an individual intends to perform a certain behaviour [12]. People’s behavioural intention determines their performance of the behaviour and it is widely used to evaluate user acceptance of technology [12, 15, 23, 24, 27]. In the case of a SMSS for renal patients, the intended behaviour is the patients’ use of this system for managing their health. In this paper it is hypothesised and tested that all the factors introduced earlier on, i.e. PE, EE, SI, FC, AF, SE, and TR, positively correlate with patients’ intention to use and therefore acceptance of the SMSS (Fig. 1).

## Methods

### Clinical setting

The data used in this study were collected in the context of a randomized controlled trial, which included an intervention group that used a SMSS during the first year post-transplantation and a control group that received usual care, which did not include self-management. The general aim of the randomised controlled trial was to investigate whether part of the post-transplantation care can be transferred to a home setting using a SMSS without compromising on the quality of care.

The study presented in this paper focuses on a survey completed by the intervention group only. The survey included a questionnaire that participants completed at the start and after 4 months into the trial.

### System description

Patients used a blood pressure meter and a creatinine device at home to measure their blood pressure and kidney function according to a fixed schedule. They were instructed to enter the measured values into a specially designed website called MijnNierInzicht (MNI), which was designed by the LUMC with help from the Dutch Organization for Applied Scientific Research (TNO) and maintained by company Bonstato. After entering their measured values, the website provided patients with an overview of their measurement history, an evaluation of their current renal function, and instructions for further actions, which could be: to continue their regular schedule, to conduct an additional measurement, or to contact the hospital. Besides the advice and monitoring function, the system included online learning modules (eLearning) providing relevant information, such as bodily functions, renal transplantation, and self-management. The system further allowed patients to record their weight, body temperature, and scheduled face-to-face and phone appointments with their doctors. The measuring devices, MNI website, and eLearning formed together the SMSS and in the survey it was referred to as the ADMIRE (Assessment of a Disease management system with Medical devices In REnal disease) system.

### Measures

A tailored renal transplant patient technology acceptance questionnaire was developed for this study. This questionnaire included several items to measure each construct included in the renal transplant patient technology acceptance model. Initial questionnaire items were based on the questionnaires reported in the literature [12, 13, 31–33, 36–38, 45–48]. These initial items were discussed in workshops with a doctor, experienced patients, and researchers in the self-management domain. This resulted in an adjusted set of items that was adapted to 1) the content of the SMSS and 2) patients’ language and knowledge. The items were all statements that had to be rated on a 7-point Likert scale with 1 for totally disagree to 7 for totally agree with the statement and a ‘not applicable’ option. Participants were asked to complete the questionnaire at the start of the study (T0) and after 4 months of using the SMSS (T1). In most cases, at T0, the questionnaire items formulation prompted for future use, while at T1 the items formulation prompted for current use. For example, the performance expectancy item PE1 at T0 was formulated as “with the ADMIRE system, I will be able to monitor my health very well myself”, while at T1 it was formulated as “with the ADMIRE system, I can monitor my health very well myself”. Still, both in T0 and T1 items related to the behavioural intention always prompted for future usage. The items were in Dutch. An English translation of the T1 questionnaire items can be found in Additional file 1. At T0, patients’ demographic data was collected, including the knowledge dimension items of the Partners in Health (PIH) scale that assesses patients’ perceived chronic condition self-management knowledge [49]. The PIH items were rated on a 9-point Likert scale from 1, for very poor, to 9 for very good. In addition, health-related information was obtained from the hospital record.

Besides collecting data related to the RTPTA model, additional data was collected related to the specific implementation of this SMSS. The additional questions focussed on satisfaction with the training given in using the system (training), patients’ options on conducting self-management through the system (self-management), contact with doctors (doctor), the time needed to use the system (time), the use of the creatinine device measuring kidney function (creatinine), the use of the blood pressure meter (blood pressure), and their feeling of conducting self-management at home (feeling, only asked at T1 as patients had to have experience with using the SMSS before being able to respond to these items, see Additional file 1). All items were rated on a 7-point Likert scale with 1 for totally disagree to 7 for totally agree with the statement.

### Procedure

Intake and training procedure differed between patients receiving a kidney from a living donor and those receiving a kidney from a deceased donor. For recipients of a living donor kidney, the transplantation procedure could be well prepared, so they received an explanation about the experiment, signed the consent form, and got access to MNI website and eLearning before the transplantation. They were explained how to use the system and were encouraged to try it themselves before transplantation. For patients who received a kidney from a deceased donor, the whole procedure was postponed to after transplantation, but was preferably arranged before discharge from the hospital. Around the day of discharge (T0), all patients were asked to complete the T0 questionnaire. At home, patients were asked to use the system regularly, according to a predefined schema for 1 year: measure and log the data daily during the first 4 weeks, every other day for week 5–9, twice a week for week 10–15, and weekly from week 16 onwards. After 4 months of using the system (T1), patients were again asked to complete the questionnaire. Both the baseline and the follow-up questionnaires were distributed in paper form.

### Participants

The intervention group consisted of renal transplantation patients who had their most recent transplantation in the LUMC. Sixty-five patients were enrolled into the trial, fifty of them responded to the questionnaire at least once, and 47 completed the 1-year trial. Eighteen patients dropped out: one patient’s transplantation was cancelled, four patients cancelled participation before start, one patient was excluded due to high level of creatinine after transplantation, two patients died before start, one patient died after start, four patients never used the system, and five patients quitted after using the system for a while. These five patients indicated a variety of reasons for this: variety in self-measured creatinine values (*n* = 3), stress caused by self-monitoring (*n* = 1), and too little benefit (*n* = 1). The profile of the participants who responded to T0 and T1 questionnaire is shown in Table 1. In both cases, 46 patients completed the questionnaires. Although these populations were not made up of the exact same responding patients, no significant differences in profile were found between the populations who responded at T0 and T1.

**Table 1:** Participant profile

Participants	T0	T1
Number	46	46
Male (%)	30 (65.22%)	29 (63.04%)
Living donor recipients (%)	40 (86.96%)	39 (84.78%)
Dialysis before transplant (%)	24 (53.17%)	23 (50.00%)
Age at transplant (sd)	51.43 (14.09)	51.87 (14.33)
Educational level		
Median (number, %)	Middle (24, 53.17%)	Middle (22, 47.82%)
Mode (number, %)	Middle (24, 53.17%)	Middle (22, 47.82%)
Number of kidney transplants		
1	43 (93.48%)	42 (91.30%)
2	3 (6.52%)	4 (8.70%)
PIH - knowledge score (sd)	7.88 (1.31)	7.96 (1.33)

### Data preparation

#### Not applicable and missing data

A distinction was made between situations where participant specifically indicated that a question was not applicable (NA) for them, or when they had left the question unanswered, i.e. missing values. The relative NA percentage, i.e., the number of NA/(the number of participants - the number of missing values) × 100% for each item was calculated. The majority of questionnaire items (77.03%) had less than 5% of the participants rated the question as NA. However, items with a relative NA percentage above 1.5 × interquartile range (4.88%) + 3^rd^ quartile (4.88%) = 12.20% were regarded as outliers [50] as apparently an unusual number of patients considered them as not applicable to their situation and were therefore not appropriate items to capture the underlying constructs across the patient sample. Twelve items (18%) turned out to be outliers and were therefore removed from the analysis, leading to the removal of the social influence construct all together and facilitating condition item 3 and 4 (all at T0 and T1, Additional file 1). For the remaining items, ‘not applicable’ was treated as missing.

There were 394 (12.71%) values missing in total. Fifteen out of fifty (30%) participants answered all the questionnaire items, and none of the items was answered by all participants. To avoid exclusion of participants and thereby biasing the analysis [51], Maximum Likelihood methods using the expectation–maximization (EM) algorithm was applied to substitute missing data of the RTPTA questionnaire items. This method produces unbiased parameter estimates with missing (completely) at random data [52]. Patients’ age, gender, type of donor, and pre-transplant status were used as predictors.

#### Behavioural intention at T0

**Fig. 2:**
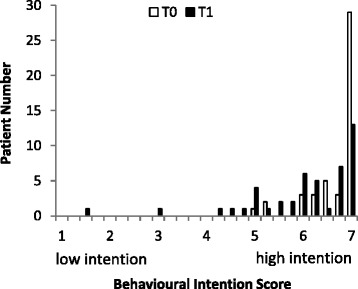
Histogram of behavioural intention measured around the discharge day (T0) and 4 months after (T1)

The behavioural intention at T0 and T1 was computed by taking the mean score of the five questionnaire items, as their Cronbach’s αs were 0.66 and 0.79, respectively. Figure 2 shows the histogram for the score at both T0 and T1. At T0 almost half (45.7%) of the patients had given the maximum score, and data showed limited variation. Variation at T1 was larger, therefore further analyses predominantly focus on data collected at T1.

### Data analysis

The data were analysed using SPSS version 22. The analyses included: Pearson correlation analyses to examine the constructs’ correlation coefficients, controlled correlation analyses to examine factors’ association with behavioural intention, *t*-tests to analyse the factors’ change between T0 and T1, and hierarchical multiple linear regression to understand how much each factor explains the observed variation between patients’ behavioural intention. To understand the possible underlying factors, correlations between patients’ characteristics, factors from RTPTA model, and behavioural intention were analysed, for which Pearson correlation, Kendall rank correlation, or point-biserial correlation were used depending on the data level. Bootstrapping procedure with 1000-sample was applied to the above analyses. This procedure is less biased by deviation from normality assumptions and by extreme values in a small sample [53, 54]. Furthermore, the analysis included Cronbach’s α and principal component analysis to examine the constructs’ reliability. As there are currently limited reports available that directly support the proposed model, the principal component analysis helped to explore how well questionnaire items of the same construct correlated with each other, and how they related with items from other constructs. Note that at a later stage when the model is more mature, the application of statistical techniques such as confirmative factor analysis would be desirable [55]. To examine the position of the rating on a 1–7 Likert scale, scores were compared with 4, which was regarded as the middle point of the scale.

## Results

### Reliability and principal component analysis

Table 2 shows the results of the reliability analysis for each construct at T1. The table also shows Cronbach’s α after items deletion for those constructs with initially low reliability level. The construct performance expectancy was split into three dimensions: 1) insight, meaning gaining insight into one’s renal condition; 2) health improvement, meaning gaining a better health status; and 3) time, meaning spending less time on outpatient appointments. As the dimension health improvement had a low reliability level, these items were excluded in further analyses.

**Table 2:** Construct reliability

Constructs	Cronbach’s α	Items to delete	Cronbach’s α if items deleted
Performance expectancy	.56		
Insight (PE1, PE2, PE3)	.73		
Health improvement (PE4, PE5, PE6)	.15	PE6	.54
Time (PE7, PE8)	.93	-	-
Effort expectancy	.67	EE3	.73
Facilitating conditions	.99		
Affect	.75		
Self-efficacy	.21	SE3, SE4	.85
Trust	.77		
Behavioural intention	.79		

A principal component analysis (PCA) was conducted on the remaining 20 independent items with orthogonal rotation (varimax). The Kaiser–Meyer–Olkin (KMO) measure verified the sampling adequacy for the analysis, KMO = 0.64, respectably above the 0.5 criterion. Two individual items had a KMO value clearly below the acceptable limit of 0.5 [56], indicating that these items share limited variance with other items. Bartlett’s test of sphericity Χ^*2*^ (153) = 662.24, *p* < .001, indicated that correlations between items were sufficiently large for PCA. The analysis resulted in five components with an eigenvalue over Kaiser’s criterion of 1. Combined they explained 73.26% of the variance. The factor loading after rotation, sampling adequacy, eigenvalue, the percentage of variance, and communality scores can be found in Additional file 2.

Although some components were mainly associated with the items from a single construct, such as performance expectancy - time dimension and effort expectancy, other components were associated with multiple constructs. The items for the constructs trust, affect, and the insight dimension of performance expectancy loaded almost together on a single component, and the same was observed for the constructs self-efficacy and facilitating conditions. This, therefore, suggested dependency between some of the constructs.

### T0 versus T1 measurement

Table 3 presents mean and standard deviation for variables of the renal transplant patient technology acceptance (RTPTA) model. Overall patients seemed positive towards using this SMSS. Paired *t*-tests comparison between T0 and T1 showed that ratings on effort expectancy, doctor, and time increased over time, while behavioural intention decreased over time. The behavioural intention had an exceptionally high score at T0, leaving mainly room for a decrease at T1.

**Table 3:** Descriptive statistics

	Constructs	T0		T1		Correlation T0 and T1	Difference T0 and T1, *t*(41)
		Mean	SD	Mean	SD		
Acceptance factors	Performance expectancy - insight	6.22**	0.80	6.04**	0.98	0.29	−1.47
	Performance expectancy - time	6.32**	0.80	6.22**	1.00	0.44*	−0.04
	Effort expectancy	6.04**	0.87	6.57**	0.68	0.25	3.36**
	Facilitating conditions	6.72**	0.54	6.75**	0.92	−0.03	0.25
	Affect	5.87**	1.00	5.90**	1.21	0.61*	−0.13
	Self-efficacy	6.06**	0.89	6.22**	1.43	0.43*	0.68
	Trust	6.10**	0.82	6.21**	0.95	0.49*	1.06
	Behavioural intention	6.63**	0.54	5.93**	1.15	0.49*	−4.50**
Different aspects	Training	6.29**	0.63	6.24**	1.06	0.29	−0.50
	Self-management	6.27**	0.85	6.35**	0.80	0.47*	0.64
	Doctor	5.80**	0.72	6.20**	0.67	0.33*	3.68**
	Time	6.38**	2.48	6.41**	0.87	0.16	2.69**
	Creatinine	6.26**	0.46	6.18**	0.77	0.29*	−0.66
	Blood pressure	6.69**	0.42	6.76**	0.35	0.34	0.85
	Feeling	-	-	4.43**	0.63	-	-

Note: H0: *μ* = 4, *p < 0.05, **p < 0.01 for bootstrapping of *t*-test, or *the 95% CI does not include 0 for bootstrapping of correlation

### Correlations

Table 4 shows correlations between the factors of RTPTA model at T1. Performance expectancy (both insight and time dimension), affect, and trust correlated significantly with behavioural intention. These factors also correlated with each other. Table 5 shows the results of controlled correlations between behavioural intention and the four (sub-)factors when controlled for the other (sub-)factors that correlated with behavioural intention. Only affect had a significant correlation with behavioural intention when controlled for other (sub-)factors.

**Table 4:** Correlations between each construct pair

	PE-insight	PE-time	EE	FC	AF	SE	TR	BI
Performance expectancy-insight	1.00	−0.02	0.19	−0.13	0.69^a^	−0.02	0.64^a^	0.32^a^
Performance expectancy-time	−0.02	1.00	0.13	0.47	0.20^a^	0.18	0.13	0.40^a^
Effort expectancy	0.19	0.13	1.00	0.01	0.30^a^	−0.02	0.27	0.13
Facilitating conditions	−0.13	0.47	0.01	1.00	0.12	0.57^a^	−0.02	0.57
Affect	0.69^a^	0.20^a^	0.30^a^	0.12	1.00	0.35^a^	0.79^a^	0.51^a^
Self-efficacy	−0.02	0.18	−0.02	0.57^a^	0.35^a^	1.00	0.15	0.37
Trust	0.64^a^	0.3	0.27	−0.02	0.79^a^	0.15	1.00	0.31^a^

Note: ^a^the 95% CI does not include 0

**Table 5:** Controlled correlation between independent factors and behavioural intention (BI)

Factors correlating with BI	Control factors	Correlation
Performance expectancy-insight	Performance expectancy-time, trust, and affect	0.07
Performance expectancy-time	Performance expectancy-insight, trust, and affect	0.36
Affect	Performance expectancy-insight, performance expectancy-time, and trust	0.39^a^
Trust	performance expectancy-insight, performance expectancy-time, and affect	−0.19

Note: ^a^the 95% CI does not include 0

### Regression analysis

Hierarchical multiple linear regression was conducted on behavioural intention. Bootstrapping with 1000 samples was again applied. First, affect, the factor that partially correlated with behavioural intention, was entered as a predictor (model 1). After this, all remaining factors that correlated with behavioural intention were entered into the model (model 2). Model 1 resulted in a significant (*F*(1, 44) = 15.80, *p* < .001) model with *R* ^*2*^ of 0.26, meaning that affect could account for 26% of the variance between patients’ usage intention, and the *p*-value suggests it was a significant predictor (Table 6). Although Model 2 has its *R* ^*2*^ improved (0.38), it was not found significantly better in explaining behavioural intention (*R* ^*2*^ change = 0.12, *sig. F* change = 0.06) than Model 1. In other words neither performance expectancy nor trust could explain patients’ behavioural intention beyond affect, which was again the only significant predictor.

**Table 6:** Model coefficients

	Coefficients					Bootstrap coefficients				
Model 1	*B*	Std. Err	*Beta*	*t*	*p*	Bias	Std. Err	*p*	95% CI	
									Lower	Upper
(Constant)	3.05	0.74		4.12	<.001	−0.21	0.96	0.002	0.50	4.26
Affect	0.49	0.12	0.51	3.98	<.001	0.03	0.15	0.001	0.31	0.90

The model was examined for possible biases caused by outliers or influential cases. First, the model fit did improve (*F*(1, 42) = 23.55, *p* < .001, *R* ^*2*^ = 0.36) after removing two outliers with standardized residuals larger than 2.58, which is more than 1% of the sample cases [56]. Secondly, influential cases were examined by calculating Cook’s distance, leverage, and DFBeta. No cases were found having Cook’s distance or standardised DFBeta larger than the recommended upper value of 1 [56]. Still two patients had their leverage value larger than the recommend upper value of 0.13, i.e. 3 × (the number of predictors + 1)/n [55]. Excluding these two patients resulted in a model with *F*(1, 42) = 16.13, *p* < .001, *R* ^*2*^ = 0.28. The original model therefore seems stable and not influenced by possible outliers or influential cases.

### Correlation with exogenous variables

The constructs affect and behavioural intention were further explore by examining correlations with patient characteristics, i.e. age, gender, donor type, educational level, the number of kidney transplants, being dialyses before transplant, and PIH - knowledge dimension. The analyses were done on paired complete cases. The analyses revealed that deceased, compared to living donor recipients, were associated with a higher Affect level, *r*_*pb*_ = .29, 95% CI [.16, .47], *n* = 42. Furthermore compared to patients that did not receive dialyses before transplant, patients that did were associated with a higher Affect level, *r*_*pb*_ = .34, 95% CI [.07, .55], *n* = 42. The analysis also revealed that female, compared to male patients, were associated with a stronger behavioural intention at T1, *r*_*pb*_ = .33, 95% CI [.16, .51], *n* = 45. No other significant correlations were found.

## Discussion

Kidney transplantation is the treatment of choice for patients with end stage renal disease, but does not free patients from needing medical care. As kidney transplant patients have to adhere to a strict medication regimen and need to be frequently monitored for signs of graft dysfunction, they are still considered chronically ill. Self-management, the process of managing symptoms, treatment, physical and psychosocial consequences by patients themselves in daily life, has been proposed to be useful when dealing with chronic illness [4]. A self-management support system (SMSS) aimed at empowering patients by giving them more control of their care process and daily activities, can help to implement self-management in daily life [5]. The current study investigated kidney transplant patients’ intention to use a SMSS and potential explaining factors.

Results show that patients were on average positive towards using the SMSS, both in advance of use and after having used the SMSS for 4 months. The behavioural intention to start or continue using the SMSS could mostly be explained by patients’ affect towards the SMSS (26% explained variance, supporting H5). The analysis also found performance expectancy on insight and on time, and trust to be correlated with behavioural intention, supporting H1 and H7 respectively. Still, these factors were not able to explain variation in behavioural intention beyond the affect factor. No support was found for the other hypotheses (H2, H3, H4, and H6). This result is different than what is usually found when using TAM or UTAUT [27], with effort expectancy being traditionally one of the most important factors explaining behavioural intention. Although 26% of explained variance is at the lower end of the range of 17% to 70% reported by other studies [27], the regression model included only one factor, which might be a reason for the relatively small *R* ^2^.

Although affect overlapped with performance expectancy to some extent, affect was the only remaining factor in the regression analysis being significantly associated with patients’ behavioural intention to continue using the system after 4 months of use. In the first few months post-transplantation, only a limited number of outpatient visits was replaced by a telephonic consult. Many patients, therefore, visited their doctors in the usual frequency, putting less need on using the system to be informed on their kidney function. The fact that there was no absolute need to use the system, contrary to what happens when an entire organisation implements a new technology and replaces the old one, might explain why affect was found to be the most important factor related to behavioural intention. When patients are ‘free’ to choose, it seems logic that emotions are crucial. Comments made by patients at the end of study participation confirm the emotional aspect. Some patients mentioned that if possible they would like to continue using the SMSS after 1 year, as it gave them a feeling of safety. Others indicated that the first year after transplantation is of most risk and as they had safely reached this milestone, they no longer felt the need to use the SMSS.

It was further found that some questionnaire items, especially the social ones such as social influence and facilitation related to the social environment, were rated as not applicable by a substantial part of the group. These participants might not have understood these questions or had not discussed the use of the system with their social environment and felt, therefore, unable to give an answer. Reformulation of these items or informing people that holding social related beliefs does not require actual discussion with the social environment might, therefore, be advisable in the future.

The main scientific contribution of the current study is that it introduced affect as a new factor explaining kidney transplant patients’ behavioural intention to use or continue using a SMSS.

In practice, the finding suggests that the emotional experience of using a SMSS should be taken into account when designing and implementing a system to be used in healthcare. Several strategies have been put forward for this, for example by empowering patients to interpret their measurements, instead of providing automatic interpretation from the system as a method to decrease patients’ stress of using the technology [57]. Furthermore, using warm colours rather than bright colours to get a calming effect, and cold colours for a more relaxing effect [58–60].

### Limitations and future research

To appreciate the study, awareness of its limitation is necessary. First, the study has a relatively small sample size considering the number of factors included in the study. Another limitation is the way of dealing with the ‘not applicable’ ratings. Although items indicated as not applicable by a substantial sub group were excluded in the analyses, others were treated as missing values, but they could have had a different meaning. A third limitation is pre-selection, as the data used in this study were derived from a group of patients that had already agreed to use the SMSS. The high intention at the beginning of the trial to use the system confirms this bias. Besides, among all 36 patients who declined to participate in the randomised controlled trial at first place, 17 patients declined because they expected additional burden and two because they expected no gain of using it, which belonged to the performance expectancy factor. Fourth, the SMSS has different components, such as the medical devices, MNI, and the eLearning modules, and the patients might have held different attitudes towards them. However, their intention to use each of these components and the corresponding influencing factors were not investigated in the questionnaire.

This work can be extended in several directions. First, enlarging the sample size would increase the statistical power, and additional research would also help to mature the model, justifying the use of more sophisticated statistical techniques such as confirmatory factor analysis, or, when including other dependent variables such as observed usage and health indicators, structural equation modelling. Second, interviewing some respondents would provide essential insights in, for example, how they interpreted the items, especially the affect items, and the rational for considering items as not applicable. This could help in the re-formulation of some items. Third, it would be interesting to include patients who would not use the SMSS to understand them as well. Another direction could be to investigate patients’ acceptance of the different components of a SMSS.

## Conclusions

This study builds a model to investigate the influencing factors for renal transplant patients to accept a self-management support system. Trust and performance expectancy could explain variation in behavioural intention of using the SMSS, but not beyond the explanation given by patients’ affect towards the system. As behavioural intention is considered an indication for system acceptance, paying attention to the emotional experience of kidney transplant patients when using an SMSS seems important for successful implementation of this kind of systems into chronic care.

## Additional files

Additional file 1: Questionnaire items. Questionnaire items used in the research at T1. (DOCX 22 kb)

Additional file 2: Summary of principal component analysis results. Results of principal component analysis of the questionnaire response. (DOCX 22 kb)

## Abbreviations

ADMIRE: Assessment a of a Disease management system with Medical devices In REnal disease
AF: Affect
BI: Behavioural intention
CKD: Chronic kidney disease
EE: Effort expectancy
ESRD: End-stage renal disease
FC: Facilitating conditions
IT: Information technology
KMO: Kaiser–Meyer–Olkin
LUMC: Leiden University Medical Centre
MNI: MijnNierInzicht
NA: Not applicable
PE: Performance expectancy
PEOU: Perceived ease of use
PIH: Partners in health scale
RTPTA: Renal transplant patient technology acceptance
SE: Self-efficacy
SI: Social influence
SMSS: Self-management support systems
TAM: The technology acceptance model
TNO: Dutch Organization for Applied Scientific Research
TPB: The theory of planned behaviour
TR: Trust
TRA: The theory of reasoned action
UTAUT: The unified theory of acceptance and use of technology
