# Supplementary material for: Renal transplant patient acceptance of a self-management support system
Source: BMC Med Inform Decis Mak. 2017 May 8;17:58. doi: 10.1186/s12911-017-0456-y (PMC5423007; doi:10.1186/s12911-017-0456-y)
Supplement: Supplementary file 1 — Questionnaire items. Questionnaire items used in the research at T1. (DOCX 22 kb) [file 12911_2017_456_MOESM1_ESM.docx]

## Questionnaire Items

### 1 Acceptance factors as formulated at T1

#### Performance expectancy

| PE1 | With the ADMIRE system, I can monitor my health very well myself. |
| --- | --- |
| PE2 | Through the ADMIRE system I understand my condition and treatment better. |
| PE3 | The ADMIRE system gives me clear insight into my current health. |
| PE4 | With the ADMIRE system, I can detect problems with my renal function earlier. |
| PE5 | I think using the ADMIRE system puts my health at lower risk. |
| PE6 | With the help of the ADMIRE system, I can play a greater role in my own medical care. |
| PE7 | I think an advantage of ADMIRE is that I can travel to the hospital less often. |
| PE8 | I think an advantage of ADMIRE is that I have more time for other activities, since I have fewer outpatient appointments. |

#### Effort expectancy

| EE1 | Working with the ADMIRE system gives me little trouble. |
| --- | --- |
| EE2 | The ADMIRE system is easy to use in my daily life. |
| EE3 | I think the use of the ADMIRE system gives no ambiguities. |
| EE4 | Learning to work with the ADMIRE system is easy for me. |

#### Social influence

| SI1 | I think my family think that I should use the ADMIRE system. |
| --- | --- |
| SI2 | I think my friends think that I should use the ADMIRE system. |
| SI3 | I think my care-givers think that I should use the ADMIRE system. |
| SI4 | I think my peer patients think that I should use the ADMIRE system. |

#### Facilitating conditions

| FC1 | My computer is good enough to use the ADMIRE system. |
| --- | --- |
| FC2 | My internet connection works perfectly. |
| FC3 | Where necessary, my family helps me to use the ADMIRE system. |
| FC4 | Where necessary, my friends help me to use the ADMIRE system. |

#### Affect

| AF1 | I find using the ADMIRE system interesting. |
| --- | --- |
| AF2-R* | I experience using the ADMIRE system as annoying. |
| AF3 | I find using the ADMIRE system pleasant. |
| AF4-R* | Using the ADMIRE system makes me feel restless. |

*R: reversed.

#### Self-efficacy

| SE1 | I can use the ADMIRE system without the help of others. |
| --- | --- |
| SE2 | I can use the ADMIRE system without the help of the ADMIRE-team. |
| SE3 | I can use the ADMIRE system as long as there is someone available to help me. |
| SE4 | I can use the ADMIRE system as long as nothing abnormal happens. |

#### Trust

| Tr1-R* | I think using the ADMIRE system puts my privacy at risk. |
| --- | --- |
| Tr2 | I am confident that the ADMIRE system works well. |
| Tr3 | I trust the information that the ADMIRE system provides me with. |
| Tr4 | I am confident that data I registered myself provides a sufficient basis for good health advice. |

*R: reversed.

#### Behavioural intention

| BI1 | I will certainly measure at the specified time points and enter the data into the ADMIRE system. |
| --- | --- |
| BI2 | I will certainly look at the overview of my measurements carefully. |
| BI3 | I will certainly follow the instructions of the ADMIRE system after entering my measurements. |
| BI4 | I will certainly first consult the eLearning module if I have medical questions. |
| BI5 | I will certainly use the planning function within the ADMIRE system to keep track of my measurement time points and my appointments with my healthcare provider. |

### 2 Different Aspects

#### Training

| The ADMIRE training teaches me useful things. |
| --- |
| I am very pleased about the introduction given by a member of the ADMIRE-team. |
| I find the online learning module very informative. |
| I got sufficient knowledge and skills to work with the ADMIRE system through the online learning module. |

#### Self-management

| With the help of the ADMIRE system, I will be able to play a greater role in my own medical care. |
| --- |
| The ADMIRE system contains lots of interesting information. |
| I find it an advantage that changes in my condition will be quickly noticed due to the frequent home measurements. |
| I find it a disadvantage that through self-measuring I will be more occupied with my kidney disease. |

#### Doctor

| I think that telephone contact with my doctor will be a full replacement for an outpatient appointment. |
| --- |
| I find it a disadvantage that when my creatinine rises I will receive an automatic notification instead of a personal message from my doctor. |
| I find it an advantage that my doctor will have direct access to my measured values. |
| I find it a disadvantage that I will see a doctor less often due to my participation in ADMIRE. |

#### Time

| I think that a disadvantage of ADMIRE is that I have to spend time on performing self-measurements. |
| --- |
| I think that a disadvantage of ADMIRE is that I have to be telephonically available at agreed time. |
| I think that a disadvantage of ADMIRE is that by self-measuring I have less time for other activities. |
| I think that an advantage of using the ADMIRE system is that I need to travel to the hospital less often. |
| I think an advantage of using the ADMIRE system is that I have more time for other activities, because I have fewer outpatient appointments. |
| I think an advantage of using the ADMIRE system is that blood samples can be taken less frequently in the hospital. |

#### Creatinine

| I am well able to use the creatinine meter. |
| --- |
| I find it an advantage that I can measure my creatinine value. |
| I find it a disadvantage that I have to prick in my finger myself. |
| I think I will find it pleasant to use the creatinine meter. |
| I will find using the creatinine meter reassuring. |
| I will find using the creatinine meter frightening. |
| I will find using the creatinine meter useful. |
| I will find using the creatinine meter frustrating. |
| I have confidence in the accuracy of the creatinine meters. |
| I will be able to carry out the self-measuring at the agreed time. |
| I will be able to assess the self-measured results. |
| I will be able to judge at what time it is important to contact the hospital. |
| I find it a disadvantage that I myself will have to react as my creatinine value increases. |
| A good self-measuring result will reassure me as much as a good outcome from the hospital laboratory. |
| I find the self-measured values match the values measured in LUMC (T1 only). |

#### Blood pressure

| I am able to use the blood pressure meter. |
| --- |
| I find it an advantage that I can measure my blood pressure. |
| I think I will find it pleasant to use the blood pressure meter. |
| I will find using the blood pressure meter reassuring. |
| I will find using the blood pressure meter frightening. |
| I will find using the blood pressure meter useful. |
| I will find using the blood pressure meter frustrating |
| I have confidence in the accuracy of the blood pressure meter. |

#### Feeling

| I am often worried about whether I have carried out the measurements correctly. |
| --- |
| After a good result of the self-measuring I am sufficiently reassured. |
| I am afraid of rejection when I measure my creatinine. |
| I regularly conduct an additional measurement to reassure myself. |
| I am not worried if I get a notification from the ADMIRE system that my creatinine has increased. |
| I am afraid of rejection when I await the results of the laboratory. |
