# Supplementary material for: Renal transplant patient acceptance of a self-management support system
Source: BMC Med Inform Decis Mak. 2017 May 8;17:58. doi: 10.1186/s12911-017-0456-y (PMC5423007; doi:10.1186/s12911-017-0456-y)
Supplement: Supplementary file 2 — Summary of principal component analysis results. Results of principal component analysis of the questionnaire response. (DOCX 22 kb) [file 12911_2017_456_MOESM2_ESM.docx]

## Summary of Principal Component Analysis Results

| Items | | Component | | | | | | sampling adequacy | Communa-lity scores |
| --- | --- | --- | --- | --- | --- | --- | --- | --- | --- |
|  |  | 1 | 2 | | 3 | 4 | 5 |  |  |
| Affect (AF3) | | **.83** | .08 | | -.04 | .25 | -.11 | .72 | .76 |
| Trust (TR4) | | **.79** | .15 | | -.09 | -.07 | .18 | .74 | .69 |
| Performance expectancy - insight (PE3) | | **.77** | -.20 | | .04 | .20 | .10 | .86 | .69 |
| Trust (TR2) | | **.77** | .13 | | .21 | .11 | .15 | .57 | .69 |
| Affect (AF2) | | **.75** | .06 | | .22 | .17 | .23 | .61 | .69 |
| Trust (TR3) | | **.72** | .02 | | -.12 | .04 | .35 | .75 | .66 |
| Affect (AF1) | | **.72** | .16 | | -.15 | -.11 | -.24 | .79 | .64 |
| Performance expectancy - insight (PE2) | | **.69** | -.11 | | .01 | -.02 | -.15 | .70 | .51 |
| Performance expectancy - insight (PE1) | | **.69** | .02 | | -.06 | .04 | .37 | .57 | .61 |
| Self-efficacy (SE1) | | .06 | **.92** | | .09 | .03 | -.01 | .73 | .85 |
| Self-efficacy (SE2) | | .15 | **.84** | | .01 | -.07 | -.02 | .58 | .73 |
| Facilitating conditions (FC2) | | -.14 | **.72** | | **.51** | .01 | .05 | .49 | .79 |
| Facilitating conditions (FC1) | | -.12 | **.71** | | **.56** | .03 | .00 | .52 | .84 |
| Affect (AF4) | | **.48** | **.52** | | .16 | .30 | .04 | .79 | .62 |
| Performance expectancy - time (PE8) | | .01 | .16 | | **.92** | -.03 | -.05 | .47 | .87 |
| Performance expectancy - time (PE7) | | .06 | .21 | | **.92** | .00 | .07 | .55 | .89 |
| Effort expectancy (EE1) | | .16 | -.06 | | .04 | **.86** | .20 | .51 | .81 |
| Effort expectancy (EE4) | | -.04 | .15 | | -.15 | **.85** | -.14 | .29 | .79 |
| Effort expectancy (EE2) | | .26 | -.12 | | .15 | **.59** | **.41** | .85 | .62 |
| Trust (TR1) | | .14 | .06 | | .01 | .12 | **.91** | .33 | .86 |
| **Eigenvalues** | **5.48** | | **3.08** | **2.48** | **2.08** | **1.54** |  |  |  |
| **% of variance** | **27.38** | | **15.40** | **12.39** | **10.41** | **7.68** |  |  |  |

*Note:* Factor loadings over .40 appear in bold.
